# Nondestructive analysis of tumor-associated membrane protein MUC1 in living cells based on dual-terminal amplification of a DNA ternary complex

**DOI:** 10.7150/thno.42951

**Published:** 2020-03-15

**Authors:** Xiaohao Liu, Dongsheng Mao, Guoli Deng, Yuchen Song, Fan Zhang, Shiqi Yang, Genxi Li, Feng Liu, Wei Cao, Xiaoli Zhu

**Affiliations:** 1 Center for Molecular Recognition and Biosensing, School of Life Sciences, Shanghai University, Shanghai 200444, P. R. China; 2 Plant Science Center, School of Life Sciences, Shanghai University, Shanghai 200444, P. R. China; 3 Oncology Department, Shanghai Ninth People's Hospital, Shanghai Jiaotong University, School of Medicine, Shanghai 201900, P. R. China; 4 Department of Oral and Maxillofacial-Head and Neck Oncology, Shanghai Ninth People's Hospital, Shanghai Jiao Tong University, School of Medicine, Shanghai 200011, P. R. China

**Keywords:** membrane protein, dual-terminal amplification, nondestructive, *in situ* fluorescence imaging, quantification

## Abstract

Non-destructive analysis of cells at the molecular level is of critical importance for cell research. At present, immunoassay-based and aptamer-based methods can achieve non-structural destructive cell analysis, but still lead to changes in cells at the molecular level. Here, we have proposed a dual-terminal amplification (DTA) strategy, which enables nondestructive analysis of membrane protein MUC1 without the effect on protein expression and cell viability in living cells.

**Methods**: A fluorophore (Cy5)-labeled DNA ternary complex consisting of three oligonucleotides is designed. It can recognize MUC1 through its aptamer region, and thus make the MUC1 of cells visible under a fluorescence microscope. When DNA polymerase is added, dual-terminal amplification is performed. One direction dissociates aptamer from MUC1, and the other direction, also known as rolling circle amplification (RCA), produces long linear DNA strands, which can be further adopted for quantitative analysis of MUC1. In this way, all reagents are removed from the surface of the cells after the analysis, which allows nondestructive analysis. We named this strategy dual-terminal amplification (DTA) analysis.

**Results**: By using the DTA analysis, both *in situ* fluorescence imaging analysis and *ex situ* fluorescence quantitative analysis of MUC1 were achieved. In addition, the aptamer-containing DNA ternary complex stays on cell surface only during the analysis and leaves the cell after the analysis is complete. The cells can be maintained in a non-interfering state for the rest of the time. So after the analysis, it is found that there are no effect on the physiological activity of cells and the expression of target protein even after two rounds of repeatable imaging and quantitative analysis.

**Conclusion**: In summary, we have successfully constructed a strategy for nondestructive analysis of membrane protein in living cells. We believe that this method provides a promising way for the analysis of the key membrane proteins of cells and the versatile utilization of precious cell samples.

## Introduction

Cell membrane is one of the most essential requirements for all organisms to exist 1. Its function is mainly mediated by proteins that form an integral part of the lipid bilayer 2. The functions of membrane proteins are diverse, including material transport, energy generation, signal transduction, etc 3. In recent years, various membrane proteins have been discovered as potential and important diagnostic targets of cancer and many other diseases in the fields of molecular biology, medical diagnosis and drug delivery 4, 5. As a typical tumor biomarker, tumor-associated membrane proteins are a kind of transmembrane glycoprotein that expressed aberrantly on tumor cells 6. It has been reported that they are targeted by 30%-40% of the marketed drugs 7. Therefore, tumor-associated membrane proteins are significant drug targets and clinical biomarker candidates 8.

Traditional detection methods of membrane proteins include immunofluorescence (IF), western blot (WB), mass spectrometry (MS) and some others 9. WB and MS methods, though can provide quantitative information of proteins, are destructive to cells. Proteins should be extracted from the lysed cells before they can be detected 10. Thus, these methods are not suitable for some specific cell analysis. For example, for cherished cell samples such as circulating tumor cells and cancer stem cells, there is a contradiction in that a sample requires both membrane protein analysis and culture for drug sensitivity testing 11. Obviously, this contradiction cannot be reconciled when the cells are destructed. So, non-structural destructive cell analysis is greatly preferred. IF technology is a powerful immunochemical technique that allows the *in situ* non-structural destructive detection of a wide variety of membrane proteins with fluorophore-modified antibodies 12-14. Though, it can be conducted on living cells, this method requires the irreversible label of target proteins with antibodies, which may unpredictably affect the activity of membrane proteins and cells 15. For example, Katsuki *et al.* demonstrated that mucin 1 (MUC1) was internalized by the binding of the anti-MUC1 antibody, from the cell surface to the intracellular region via the macropinocytotic pathway 16.

Recently, aptamer-based strategies have been widely used in the analysis of membrane proteins 17-20. Aptamers are single-stranded DNA or RNA oligonucleotides which can fold into specific three-dimensional conformation to bind targets 21. A variety of aptamers have been screened out to bind membrane proteins with high affinity and specificity (e.g. the dissociation equilibrium constant (Kd) of MUC1 aptamer is about 100 nM) 22-24. However, like the methods based on immune recognition, aptamer can still cause a change in the expression of the target proteins 25. For example, AS1411 is a DNA aptamer which can target nucleolin (a protein which is overexpressed in many tumor types) and inhibit its expression 26, 27. Thus, nondestructive analysis of tumor-associated membrane proteins in living cells is still a vital problem to be solved 28.

**Scheme 1:**
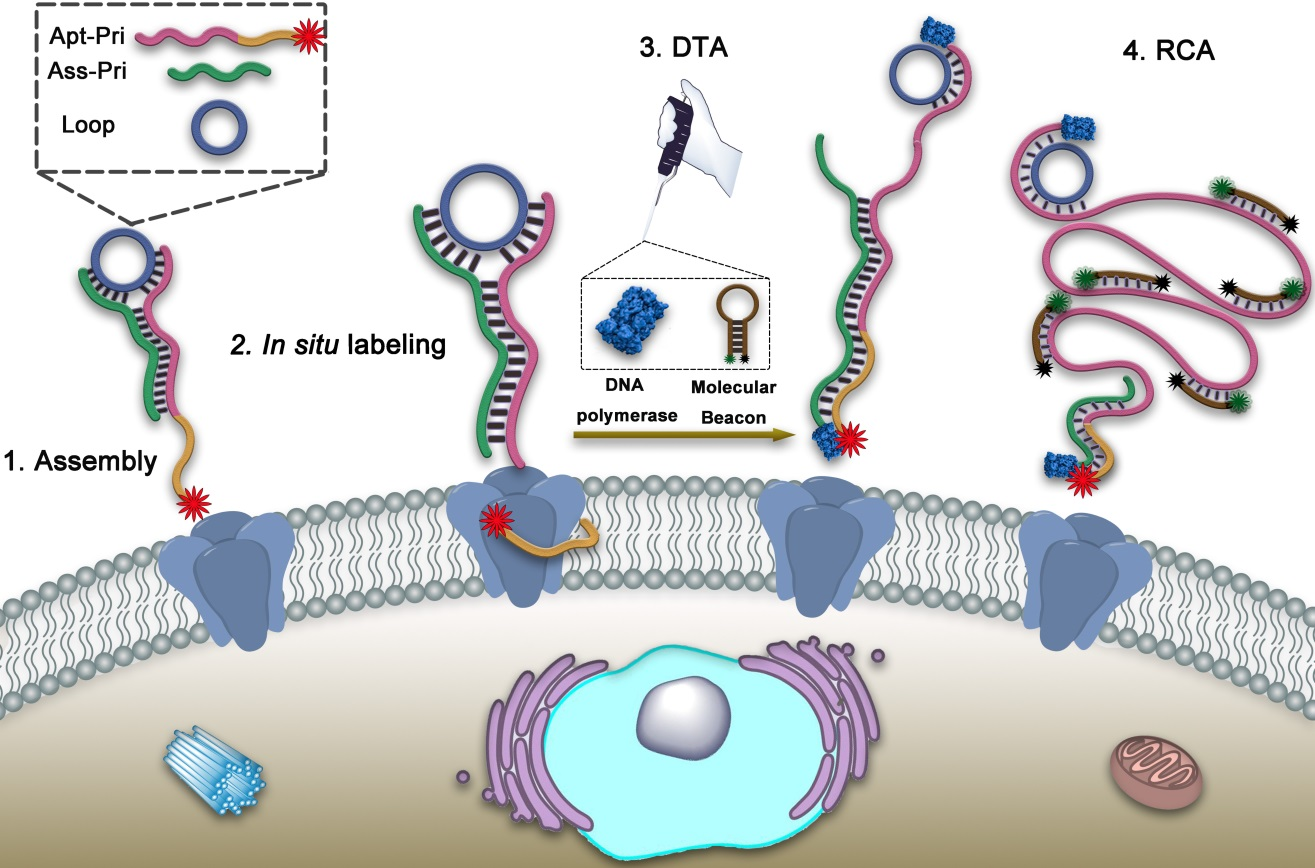
Schematic illustration of nondestructive assay of tumor-associated membrane proteins based on a dual-terminal amplification (DTA) strategy.

To address this problem, here we propose a dual-terminal amplification (DTA) strategy based on DNA ternary complex for nondestructive analysis of tumor-associated membrane proteins in living cells. MUC1, a transmembrane mucin glycoprotein that has been shown to be highly expressed in malignant epithelial cells and is considered a tumor-associated membrane protein, was adopted as a model target 29-32. As illustrated in Scheme 1, the DNA ternary complex is consisted of three single-stranded DNA by Watson-Crick base pairing, which including a circular DNA template (Loop), an aptamer-primer (Apt-Pri) and an assistant primer (Ass-Pri). The Apt-Pri is designed to include two sections, the yellow part is the aptamer of the target protein, which is adopted specifically recognize and bind to MUC1 and the red part is the primer of rolling circle amplification (RCA). At the 5'- terminal of Apt-Pri, a red fluorophore (Cy5) is labeled, which is used for the location analysis of MUC1. The Ass-Pri and the Loop can hybridize with the Apt-Pri, which can form the structure of DNA ternary complex and further initiate DTA. The formation of this structure is conducted in solution through the strict complementary nature of the base pairs. Then the DNA ternary complex can bind to the target protein by the high affinity of aptamer. When the DNA polymerase (Klenow Fragment 3'→5', exo^-^) and the molecular beacon (MB) are added, the Apt-Pri and Ass-Pri can extend respectively under the catalysis of the DNA polymerase. The extension of Ass-Pri releases DNA ternary complex from target membrane protein and the extension of Apt-Pri, that is, rolling circle amplification (RCA), releases large quantities of tandem repeat templates, which can be specifically recognized by MB, to solution for quantification. MB is labeled with a fluorophore (FAM) at its 5'-terminal and a quencher (BHQ1) at its 3'-terminal, respectively. And the fluorescence of FAM can be recovered in the presence of RCA products. Thus, the amplified fluorescent signals from the reaction supernatant can be detected by a fluorescence spectrophotometer, and is expected to have a correlation with the amount of target proteins. In this strategy, imaging and quantification analysis can be conducted one after another so as to provide both location and quantification information of specific target proteins. Meanwhile, the goal of nondestructive analysis of tumor-associated membrane proteins is achieved based on DTA method.

## Experimental section

### Materials and reagents

Loop was purchased from Takara Biotechnology (Dalian, China). All other DNA oligonucleotides were synthesized by Sangon Biotech Co., Ltd. (Shanghai, China) and purified by high-performance liquid chromatography (HPLC). All DNA sequences are listed in Table S1. DNA polymerase (Klenow Fragment, 3'→5' exo^-^) was purchased from Thermo Fisher Scientific. Hoechst 33342 and 4', 6-diamidino- 2-phenylindole (DAPI) were purchased from Jiangsu Kaiji Biotechnology Co., Ltd. Dulbecco's modified Eagle's medium (DMEM), fetal bovine serum (FBS), antibiotics (penicillin-streptomycin-nystatin solution) and 0.25% EDTA-trypsin were purchased from Biological Industries (Beit Haemek, Israel). 4% paraformaldehyde (PFA) was purchased from BBI (BBI Life Sciences Corporation, Shanghai, China). Chlorpromazine (CPM) was purchased from Aladdin Reagent Co., Ltd. Bovine serum albumin (BSA) were purchased from Sinopharm Chemical Reagent Co., Ltd. Benzyl-α-GalNAc was purchased from Sigma-Aldrich. Annexin V-FITC Apoptosis Detection kit was purchased from BD Biosciences (New Jersey, USA). Cell Counting Kit-8 (CCK-8) was purchased from Dojindo Molecular Technologies, Inc. (Japan). Anti-MUC1 antibody (ab45167), anti-GAPDH antibody (ab181602), goat anti-rabbit IgG H&L (Alexa Fluor® 488) (ab150077) and goat anti-rabbit IgG H&L (HRP) (ab97051) were purchased from Abcam (Cambridge, UK). Laser confocal glass-bottomed dishes were purchased from NEST Biotechnology Co., Ltd (Wuxi, China). HeLa (human cervical cancer cell), MCF-7 (human breast cancer cells), A549 (human lung cancer cells) and HepG2 (human hepatocellular carcinoma cell) cell lines were all obtained from the Cell Bank of Chinese Academy of Sciences (Shanghai, China). Human breast cancer (BC) cells and human hepatocellular carcinoma (HCC) cells acquired from *in vivo* tissues and cultured *in vitro* were taken from two cancer patients admitted to Shanghai Ninth People's Hospital. The study was approved by the hospital Research Ethics Committee. All subjects gave written informed consent to participate.

### Cell culture

All the cells were cultured in DMEM containing 10% FBS, 100 U/mL penicillin, 100 μg/mL streptomycin, and 12.5 U/mL nystatin, and incubated in a humidified atmosphere of 5% CO_2_ at 37 °C. Cells were collected at the end of the log phase and then counted with an automated cell counter for the following experiments.

### Aptamer labeling

Firstly, aptamers were heated to 95 °C for 5 min to denature and then cooled slowly to room temperature to form the specific aptamer structure in 1× PBS (pH 7.4). Cancer cells were seeded in laser confocal glass-bottomed dishes and incubated at 37 °C for 24 h for better cell attachment. In order to prevent the endocytosis of aptamer, 100 μM of CPM (Chlorpromazine) was added into the medium and incubated with the cells for 30 min at 37 °C in a cell culture incubator. After removing the medium containing CPM by washing gently twice with 1× PBS, the cells were incubated with aptamer-protein binding buffer (DMEM containing 1 mg/mL BSA, 10% FBS, 4.5 g/L glucose, 5 mM MgCl_2_) containing 150 nM aptamer at 37 °C for 60 min in a cell culture incubator. After the reaction finished, aptamer- protein binding buffer containing aptamers were removed and washed gently twice with 1× PBS. The cell nucleuses were stained with Hoechst 33342 for 10 min and imaged using a LSM 710 confocal laser scanning microscope (Zeiss, Germany).

### Dual-terminal amplification

The mixture of Apt-Pri (aptamer-primer), Ass-Pri (assistant primer) and Loop (circular DNA template) were heated at 95 °C for 5 min and cooled to room temperature slowly. Then the hybridization of Apt-Pri, Ass-Pri and Loop was performed in 100 μL of aptamer-protein binding buffer (DMEM containing 1 mg/mL BSA, 10% FBS, 4.5 g/L glucose, 5 mM MgCl_2_) containing 150 nM of Apt-Pri, Ass-Pri and Loop at 37 °C for 60 min to form DNA ternary complex. In order to prevent the endocytosis of aptamer, 100 μM of CPM was added into the medium and incubated with the cells for 30 min at 37 °C in a cell culture incubator. After removing the medium by washing gently twice with 1× PBS, the DNA ternary complex was incubated with cells at 37 °C for 60 min. Cells were washed twice with PBS and imaged under a confocal laser scanning microscope. 100 μL of a reaction system containing 2 μL of 10mM dNTPs, 2μL of 5 U/μL DNA polymerase, 10 μL of 10× reaction buffer, 5 μL of 10 μM molecular beacon and 81 μL of DMEM were incubated with cells for 2 h to allow the proceeding of the dual-terminal amplification. Afterward, 80 μL of the reaction solution was collected. And, the fluorescence of the molecular beacon hybridized with the amplification products in the solution was measured using an FL-7000 fluorescence spectrometer.

### Immunofluorescence

Firstly, 4% paraformaldehyde was used to fix cells for 10 min at room temperature. Then incubated cells with 1% BSA in PBST (PBS + 0.1% Tween 20) for 60 min to block unspecific binding of the antibodies. After being washed with PBS, the cells were incubated in the diluted antibody (1:500 dilution) in 1% BSA in PBST overnight at 4 °C. Decanted the solution and washed the cells three times with PBS, 5 min each wash. The cells were incubated with Alexa Fluor 488-labeled secondary antibody (1:1000 dilution) in 1% BSA for 1 h at room temperature in the dark. After being washed three times with PBS for 5 min each in the dark, the cells were stained with DAPI for 10 min and imaged using a LSM 710 confocal laser scanning microscope (Zeiss, Germany).

### Fluorescent measurements

In the presence of RCA products, the BHQ (Black Hole Quencher)-quenched fluorescence of the fluorophore FAM at the terminal of the molecular beacon were recovered. The fluorescence spectra of FAM were collected between 500 nm and 600 nm using a maximal excitation wavelength of 488 nm. The peak fluorescence at 520 nm was recorded for quantification. All the fluorescent measurements were carried out using an F-7000 Fluorescence Spectrophotometer (Hitachi, Japan).

### Gel electrophoresis analysis

Polyacrylamide gel electrophoresis was used for the characterization of the different DNA structures. A volume of 8 μL of the samples together with 2 μL 5× loading buffer were loaded onto a 15% non- denaturing polyacrylamide gel. The electrophoresis experiments were carried out in 1× Tris-boric acid-EDTA (TBE) at 120 V for 90 min. The imaging of the gel was performed using a Gel Doc XR Imaging System (Bio-Rad, USA).

Agarose gel electrophoresis was performed for analysis of the RCA products. Likewise, a volume of 4 μL of the samples together with 1 μL 5× loading buffer were loaded onto a 2% agarose gel. The electrophoresis experiments were carried out in 1× TAE at 120 V for 30 min. The imaging of the gel was performed using a Gel Doc XR Imaging System (Bio-Rad, USA).

### Western blot analysis

Membrane proteins lysates were extracted from cells using RIPA (radio- immunoprecipitation assay) buffer containing 1% Phenylmethanesulfonyl fluoride (PMSF) on ice. After quantification with BCA protein kit, 10 μL of the proteins were separated by standard 12% SDS-PAGE gel and electro-transferred onto a polyvinylidene difluoride (PVDF) membrane. Then, the PVDF membrane was blocked with 2% (w/v) BSA in TBS with tween 20 (TBST) for 2 h at room temperature, and washed with TBST three times, 5 min each. Incubate the membrane with rabbit monoclonal antibody to MUC1 (mucin-1) (1:1000 dilution) and GAPDH (glyceraldehyde-3-phosphate dehydrogenase) (1:5000 dilution) antibody respectively at 4 °C overnight. Here, GAPDH was used as an internal control. After being washed with TBST with three times, the PVDF membrane was incubated with a secondary anti-rabbit antibody conjugated to horseradish-peroxidase (1:5000 dilution) for 1 h at room temperature. Finally, the western blot signals were performed with ECL chemiluminescence kit (China) and detected by a ChemiDocTM XRS Plus luminescent image analyzer (Bio-Rad, USA).

### Flow Cytometry Assay

The cell apoptosis assay was performed by FITC Annexin V Apoptosis Detection Kit according to the recommended protocol. The cancer cells were seeded at a density of 1 × 10^5^ cells/well in a 6-well plate and kept in the incubator for at least 24 h for better cell attachment. After being treated with reaction buffer and DNA ternary complex, the cells were collected through trypsin digestion and resuspended in 1× Binding Buffer at a concentration of 1 × 10^6^ cells/mL. Then 5 μL of FITC Annexin V and 5 μL of PI were added per 100 μL of resuspended cells (1 × 10^5^ cells). After vortexing gentlely, the cells were incubated for 15 min at room temperature in the dark. Finally, 400 μL of 1× Binding Buffer was added to each tube before analyzing. The fluorescence intensity of the cells was detected by a MoFlo XDP Flow Cytometer/Sorter (Beckman Coulter, Inc., Brea, USA).

### Cell viability assay (CCK-8 assay)

The cell viability was assessed by Cell Counting Kit-8 (CCK-8) assay according to the manufacturer's instructions. In detail, the cells were seeded at a density of 1 × 10^4^ cells/well in a 96-well plate and kept in the incubator for at least 12 h for better cell attachment. The medium alone (Ab) and the medium with cells (Ac) served as the blank group and control group, respectively. The medium with cells and analyzed by DTA served as test group (As). After 48 h incubation, 10 μL of CCK-8 reagent was added to each well and cells were continuously incubated at 37 °C for 2 h. Finally, the OD value was evaluated by absorbance measurements at 450 nm on a microplate reader. The assay was repeated three times and the cell viability was calculated as following:

Cell viability (%) = [(As-Ab) / (Ac-Ab)] × 100%

### Isothermal titration calorimetry (ITC)

ITC experiment was performed at 37 °C on high-feedback mode with a stirring speed of 437 r.p.m. (Malvern VP-ITC) and a filter time of 20 s. The first injection was 5 μL followed by 10 μL per injection. Long-per-injection delays (600 s) and 120 s initial delay were used in order to establish a flat baseline. Solution of Apt-Pri were used as titrant with the concentration of 400 nM to titrate MUC1 mimic (concentration of MUC1 mimic is 5 μM). The obtained data was fitted with one set binding model.

### Statistical analysis

GraphPad Prism (GraphPad Software, La Jolla, CA) was used for statistical analysis. Results were presented as mean ± S.D. All comparisons between two groups were performed with Student's t-test. Differences were considered statistically significant when p < 0.05.

## Results and Discussion

### Feasibility of DTA based on DNA ternary complex

Initially, assembly of the DNA ternary complex is verified by native polyacrylamide gel electrophoresis (PAGE). A complementary sequence of aptamer section of the Apt-Pri, termed MUC1 mimic, was used as the mimic of MUC1 (Figure S1). From the results of native PAGE, we demonstrate that the DNA ternary complex (lane 12) and the DNA ternary complex binding with MUC1 mimic (lane 13) can be successfully constructed. In order to study the feasibility of RCA *in vitro*, a 2% agarose gel was used to analyze the amplification reaction (Figure S2). RCA products in lane 8 (with DNA polymerase) can be obviously observed, which demonstrate that the DNA ternary complex could conduct RCA with DNA polymerase. For better verify the detachment of MUC1 mimic from DNA ternary complex, a MUC1 mimic modified with a fluorophore (FAM) at the 5'- terminal is employed (Figure S3). Results show that the MUC1 mimic can detached from the Apt-Pri during RCA reaction. The dissociation equilibrium constant (Kd) of Apt-Pri and the extended parted of Ass-Pri (MUC1 mimic) is evaluated to be 2.78 ± 1.44 nM (Figure S4), which is lower than that of the aptamer with MUC1 protein (ca. 100 nM 28, 29), suggesting the extended parted of Ass-Pri could rival and detach MUC1 protein from its aptamer. These above results indicate that DTA method is successfully developed, which can be used for further analysis on cells.

**Figure 1:**
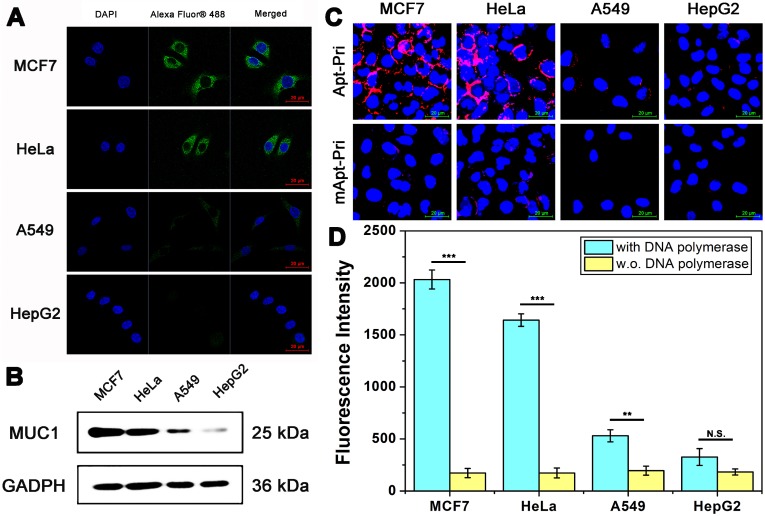
** Imaging and qualitative detection of MUC1 in MCF-7, HeLa, A549, and HepG2 cells. (A)** Confocal laser scanning microscope images of MUC1 with immunofluorescent assay using anti-MUC1 antibody. The cells had been fixed and permeated (dead cells). **(B)** Western blot results of MUC1 expression level. **(C)** Confocal laser scanning microscope images of MUC1 labeled with Cy5-labeled Apt-Pri or mApt-Pri for 60 min of incubation. **(D)** The fluorescent signals of FAM-labeled molecular beacon that were released into the supernatant after the DTA analysis by using fluorescence spectrometer. Ex: 488 nm, Em: 520 nm. The data represent the mean ± SD (error bars) of triplicate experiments. N.S., no significance. *, p<0.05. **, p<0.01. ***, p<0.001.

Next, the DTA was performed on four different cell lines (MCF7, HeLa, A549 and HepG2). As shown in Figure 1A, the expression level of MUC1 in these four cell lines was firstly studied by immunofluorescence (IF). The fluorescence intensity of MUC1 on the surface of MCF-7 cells and HeLa cells are higher than that of A549 cells. Whereas nearly no fluorescent signals are found on the surface of HepG2 cells, which indicate that the expression level of these cell lines differs with a gradient of MCF-7 > HeLa > A549 > HepG2, and only HepG2 cells are MUC1 negative. In order to further verify the expression level of MUC1, western blot analysis is employed (Figure 1B). Results show that the expression level of these cell lines coincides well with the results of IF. The binding ability of aptamer to MUC1 is then conducted to detect the binding ability between aptamer and target protein using Apt-Pri labeled with a red fluorophore (Cy5) (Figure 1C). The Apt-Pri containing aptamer sequence can specifically binds to MUC1, but the mApt-Pri (mismatched Apt-Pri, a random DNA sequence) can not. Like the results of IF, there is a positive correlation between the fluorescence intensity of MUC1 on the surface of cells and the MUC1 expression level. Furthermore, tumor cells with DTA can produce quantitative fluorescent signals that can be collected from reaction supernatant for the detection of tumor-associated membrane proteins (Figure 1D). These amplified fluorescent signals can be collected using a fluorescence spectrophotometer, and results indicate that there is a positive relationship between the amount of tumor-associated membrane proteins and the fluorescent signals. The above results constantly prove that MUC1 expression levels of these four cell lines differ apparently with a gradient of MCF-7 > HeLa > A549 > HepG2. Therefore, these three cell lines (MCF-7 cells, HeLa cells and A549 cells) are selected as positive experimental group, while HepG2 cells are selected as negative control for the following experiments.

### Optimization of experimental conditions

**Figure 2:**
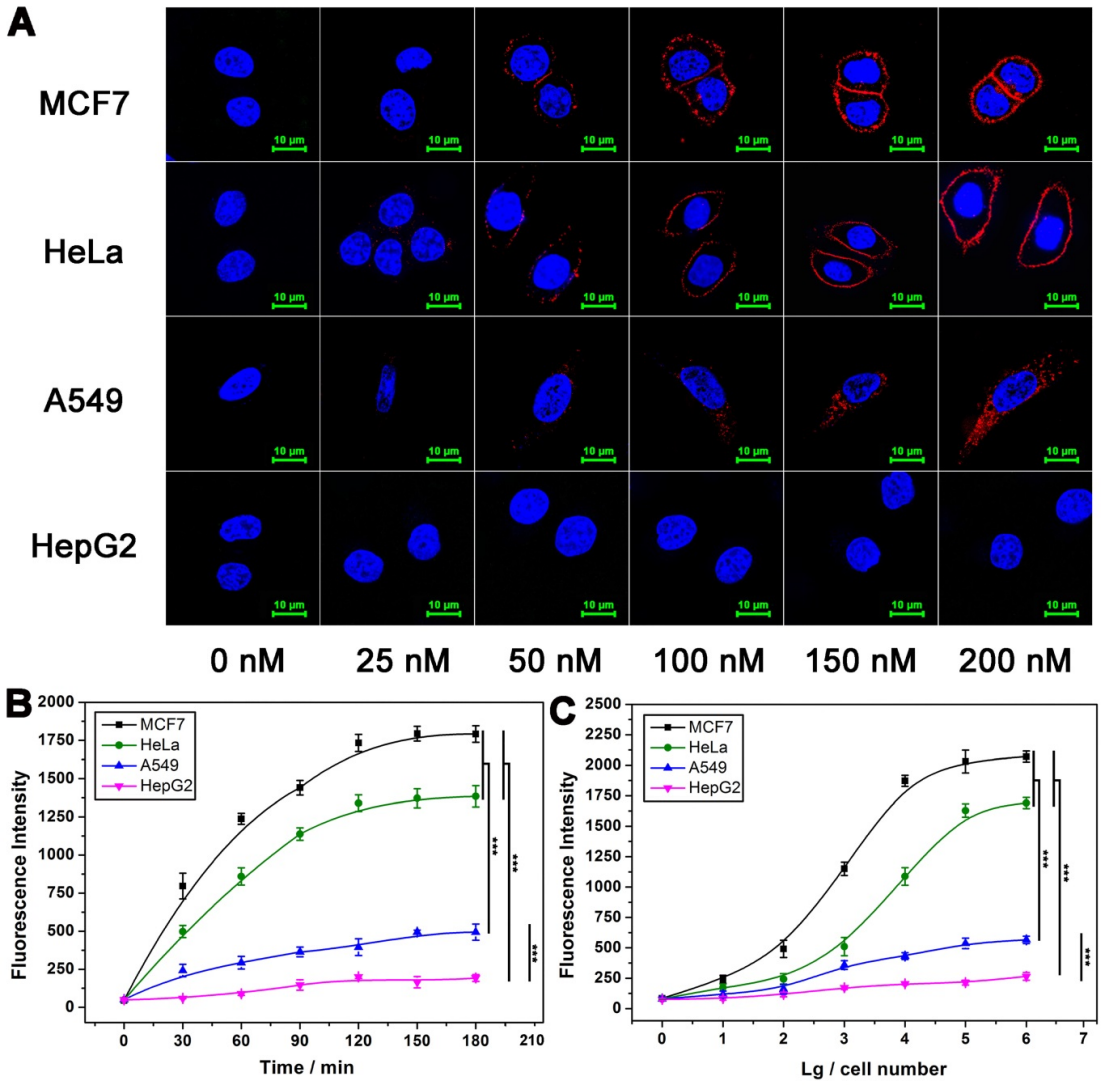
** Effect of some conditions on the performance of DTA analysis. (A)** Confocal laser scanning microscope images of MUC1 after cells were incubated with different concentrations of Cy5-labeled Apt-Pri. **(B)** Optimization of polymerase amplification time of DTA analysis by monitoring the fluorescence intensity of FAM-labeled molecular beacon that were released into the supernatant after the DTA analysis. Ex: 488 nm, Em: 520 nm. **(C)** Fluorescence quantitative signals of MUC1 with various cell numbers. The signals were also obtained from FAM-labeled molecular beacon. The data represent the mean ± SD (error bars) of triplicate experiments. N.S., no significance. *, p<0.05. **, p<0.01. ***, p<0.001.

DNA ternary complex is formed on the basis of binding effect between Apt-Pri and target protein. On this basis, the concentration of Apt-Pri and the binding time are firstly optimized. As shown in Figure 2A, the fluorescence intensity of MUC1 on the surface of cells increases with the increase of Apt-Pri concentration and starts to saturate when the concentration of Apt-Pri reaches 150 nM. Consequently, 150 nM is adopted as optimal concentration of Apt-Pri. Next, the incubation time of Apt-Pri with cells was investigated and 60 min can be adopted as optimal reaction time for the binding of Apt-Pri to MUC1 (Figure S5). Subsequently, in the process of DTA, as shown in Figure S6, the fluorescence intensity of MUC1 on the surface of cells with DNA polymerase shows a significant decrease and almost disappeared after 120 min. Because the fluorescence is originated from Cy5-labeled Apt-Pri, the disappearance of its fluorescence means that the Apt-Pri has detached from MUC1. Meanwhile, figure 2B shows that the fluorescence intensity of reaction supernatant increases with the increment of time and starts to saturate at 120 min. Therefore, 120 min is adopted as an optimized time of DTA reaction in the following experiments. Quantification of membrane proteins with different cell numbers is then conducted using the fluorescent signals from the reaction supernatant. As shown in Figure 2C, the fluorescence intensity of reaction supernatant increases with the increase of cell numbers. The fluorescence signal has the highest sensitivity between 100 and 100,000 cells, so this range can be used as a range for quantitative detection. It can be seen that cells less than 10 are enough to provide quantitative results based on DTA assay. Compared to traditional WB assay, DTA assay do not need tedious operation and a large number of cells (less than 10 cells for DTA assay and at least 1×10^5^ cells for WB).

### Response of DTA to the expression level of MUC1

**Figure 3:**
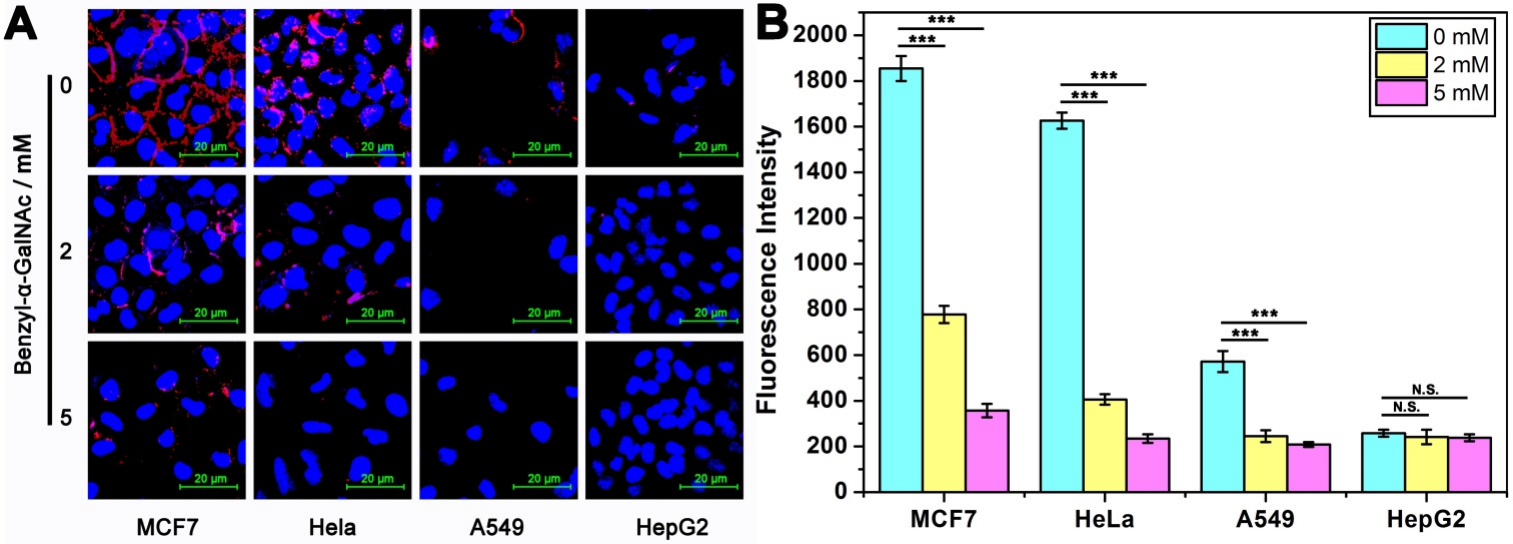
** Imaging and quantitative detection of MUC1 in tumor cells which had been pre-treated with 0/2/5 mM MUC1 inhibitor (benzyl-α-GalNAc). (A)** Confocal laser scanning microscope images of MUC1 labeled with Cy5-labeled Apt-Pri. **(B)** Quantitative detection of MUC1 by monitoring the fluorescence intensity of FAM-labeled molecular beacon that were released into the supernatant after the DTA analysis. Ex: 488 nm, Em: 520 nm. The data represent the mean ± SD (error bars) of triplicate experiments. N.S., no significance. *, p<0.05. **, p<0.01. ***, p<0.001.

Next, to study the changes in the expression level of MUC1 on cancer cells, a specific inhibitor of MUC1, benzyl-α-GalNAc that had been proved to be non-toxic to cells (Figure S7), was investigated. Firstly, the fluorescence intensity on the surface of cells was studied (Figure 3A). After incubated with the medium containing different concentrations of inhibitors (0 mM, 2 mM and 5 mM) for 60 h, we found that the fluorescence intensity of MUC1 on the surface of cells after treatment with 2 mM and 5 mM inhibitors are significantly decreased compared to control group (without inhibitor treatment). Furthermore, we also studied the fluorescence intensity of the reaction supernatant after DTA analysis (Figure 3B). As expected, as the concentration of inhibitors increases, the fluorescence intensity of the reaction supernatant of all the four cell lines are significantly decreased until close to the background. These results coincide well with each other, and they all show that MUC1 inhibitor significantly affected the expression level of MUC1 of the cells, and DTA assay is able to respond the change in MUC1 expression level sensitively.

### Repeatable analysis of MUC1 based on DTA assay

**Figure 4:**
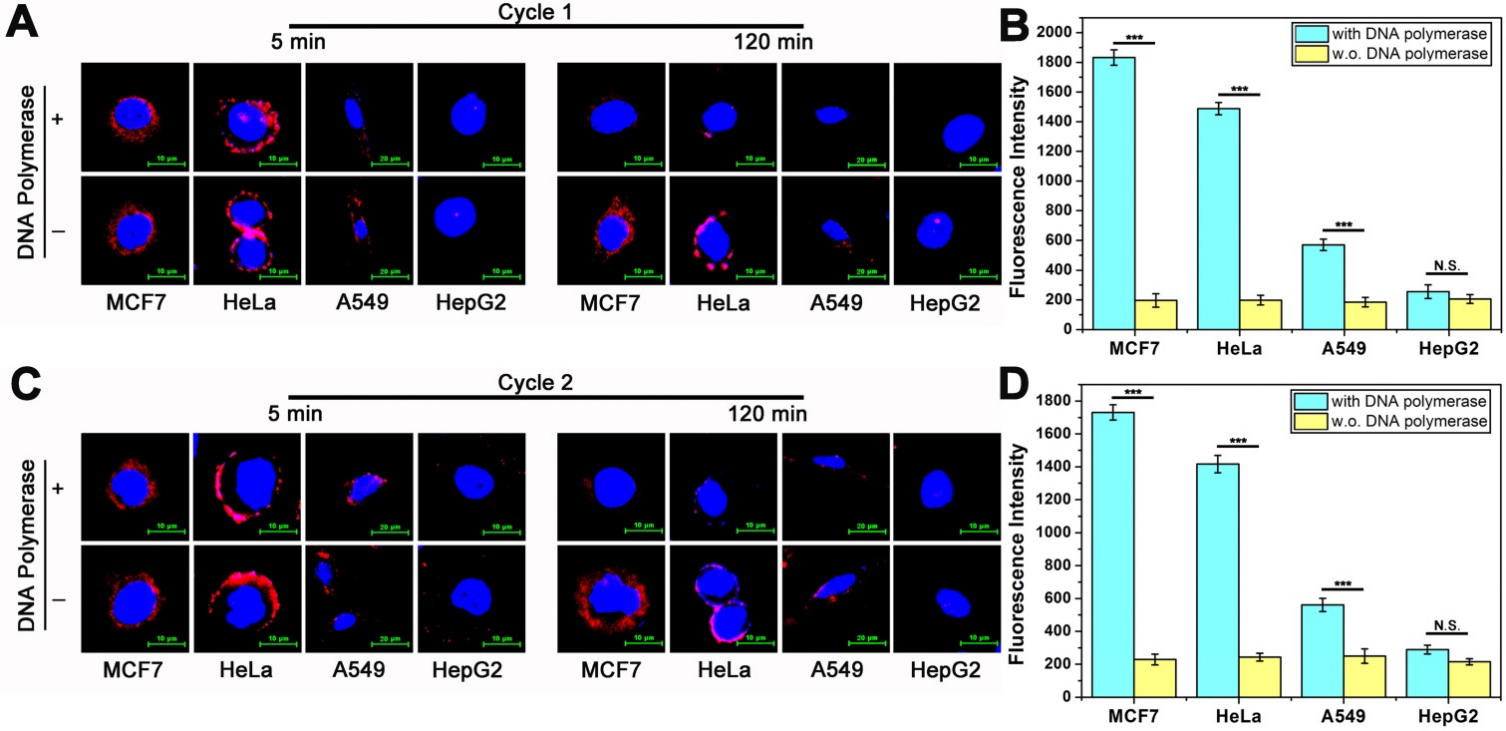
** Repeatable imaging and quantification of MUC1 in tumor cells based on DTA method. (A)** Confocal laser scanning microscope images of MUC1 labeled with Cy5-labeled Apt-Pri in the first round of DTA analysis. **(B)** The fluorescent signals of released FAM-labeled molecular beacon after the first round of DTA analysis. Ex: 488 nm, Em: 520 nm. **(C)** Confocal laser scanning microscope images of MUC1 labeled with Cy5-labeled Apt-Pri in the second round of DTA analysis. **(D)** The fluorescent signals of released FAM-labeled molecular beacon after the second round of DTA analysis. Ex: 488 nm, Em: 520 nm. The data represent the mean ± SD (error bars) of triplicate experiments. N.S., no significance. *, p<0.05. **, p<0.01. ***, p<0.001.

DTA assay enables localization and quantification analysis of membrane proteins. Because DTA assay is operated in a medium environment and membrane proteins maintained in a non-interfering state after analysis. Therefore, repeatable analysis and nondestructive analysis are believed to be two of the most important features of DTA-based assay. To address the feature of repeatable analysis, here, we investigate the fluorescence intensity of MUC1 on the surface of cells for imaging and the fluorescent signals from reaction supernatant for quantification with two rounds of DTA analysis. We firstly detect the fluorescence intensity of MUC1 on the surface of cancer cells. All samples were divided into two groups: DTA assay treated sample group (with DNA polymerase) and corresponding control group (w.o. DNA polymerase). The operation of the first round of experiments was as follows. After being incubated with DNA ternary complex for 60 min, cancer cells were washed gently with PBS and imaged by confocal laser scanning microscope, as shown in Figure 4A, which is consistent with previous results (5 min, with and w.o DNA polymerase). Subsequently, the fluorescent signal on the surface of the cells are almost disappeared in DTA treated group (120 min, with DNA polymerase). On the contrast, there are no significant change in the fluorescent signal in control group (120 min, w.o. DNA polymerase). After being incubated at 37 °C for 120 min, the fluorescence intensity of the reaction supernatant of all the samples were measured. As shown in Figure 4B, the result coincides well with the imaging results of Figure 4A and previous quantification results (Figure 2B). The different performance between with and w.o. polymerase group also proves that the decrease in fluorescent signal on the surface of membrane and the increase of fluorescence intensity in the reaction supernatant solution is derived from efficient amplification instead of false positive. After the first round of analysis, the cells were recultured in the incubator for further 2 hours for subsequent second round of analysis. The operation of second round analysis is the same as that of the first round. As shown in Figure 4C and Figure 4D, the experiment results are coherent with the previous. These above experimental results show that the goal of repeatable imaging and quantification of membrane proteins is achieved. It is worth noting that, these two rounds of analysis have good repeatability, which demonstrates that nondestructive analysis probably achieved during the analysis.

### DTA-based nondestructive analysis

**Figure 5:**
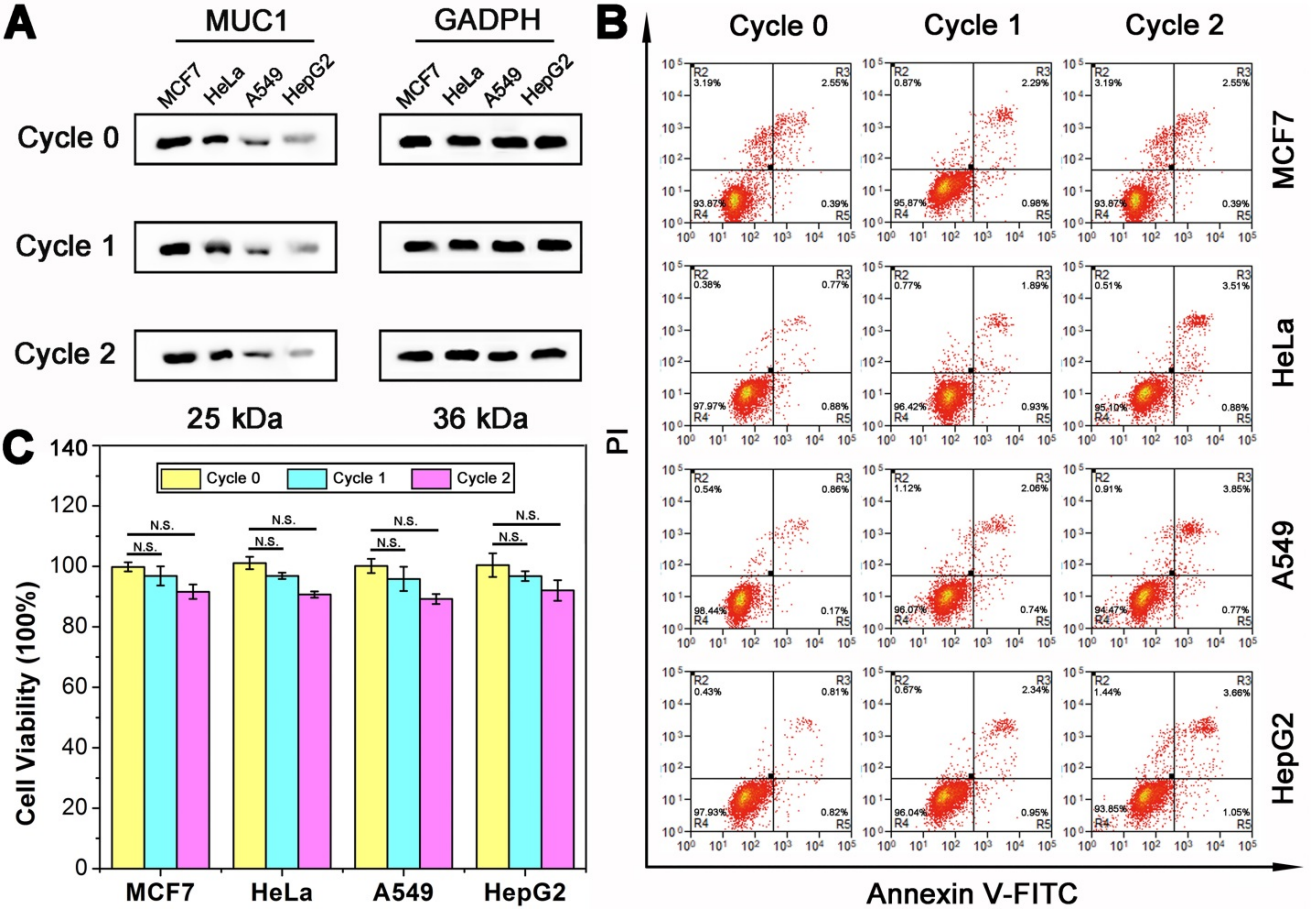
** Cell viability and protein expression after DTA analysis. (A)** Western blot results of MUC1 expression before (Cycle 0) and after one (Cycle 1) or two (Cycle 2) rounds of DTA analysis. **(B)** Flow cytometry analysis of tumor cells apoptosis with 0/1/2 rounds of DTA analysis using an Annexin V-FITC Apoptosis Detection kit. **(C)** CCK-8 analysis of cell viability with 0/1/2 rounds of DTA analysis. The data represent the mean ± SD (error bars) of triplicate experiments. N.S., no significance. *, p<0.05. **, p<0.01. ***, p<0.001.

Subsequently, to demonstrate the nondestructive feature of DTA assay, the effect of DTA assay on cell viability should be analyzed. As shown above, during the DTA-based analysis of MUC1, the reaction system as well as the gentle treatments are expected to impact little on the physiological activity of both the cells and membrane proteins. Therefore, the cells can be adopted for further experiments, such as culture, testing of drug susceptibility, other studies of membrane proteins, *etc*. In order to verify the effect of DTA-based analysis method on membrane protein, the level of MUC1 expression after two rounds of DTA analysis was first studied using western blot assay. As shown in Figure 5A, the expression level of MUC1 before and after two rounds of DTA analysis (Cycle 0, Cycle 1 and Cycle 2) was investigated. Results show that the expression level of MUC1 is almost not changed even after two rounds of DTA analysis. Then, the study of cell apoptosis status was conducted using flow cytometry with an Annexin V-FITC Apoptosis Detection kit (Figure 5B). Over 95% and 93% cell viability were observed in the Cycle 1 and Cycle 2 group, respectively. Which indicates that there is no significant distinction of the cell viability before and after DTA-based analysis. In addition, the cell viability with two rounds of DTA analysis was measured immediately or after 24-72 hours of culture by CCK-8 assay. As shown in Figure 5C and Figure S8, no significant difference in cell viability is observed before and after DTA analysis. In comparison, the effects of aptamer and antibody on cells were further investigated. The results showed that after incubating the cells with Apt-Pri or anti-MUC1 antibody for 72 hours, the expression of MUC1 was reduced to some extent, while the cell viability was hardly affected (Figure S9 and Figure S10). This phenomenon is consistent with some literatures and shows that both antibody and aptamer have a certain effect on cells at the molecular level 16, 26.

**Figure 6:**
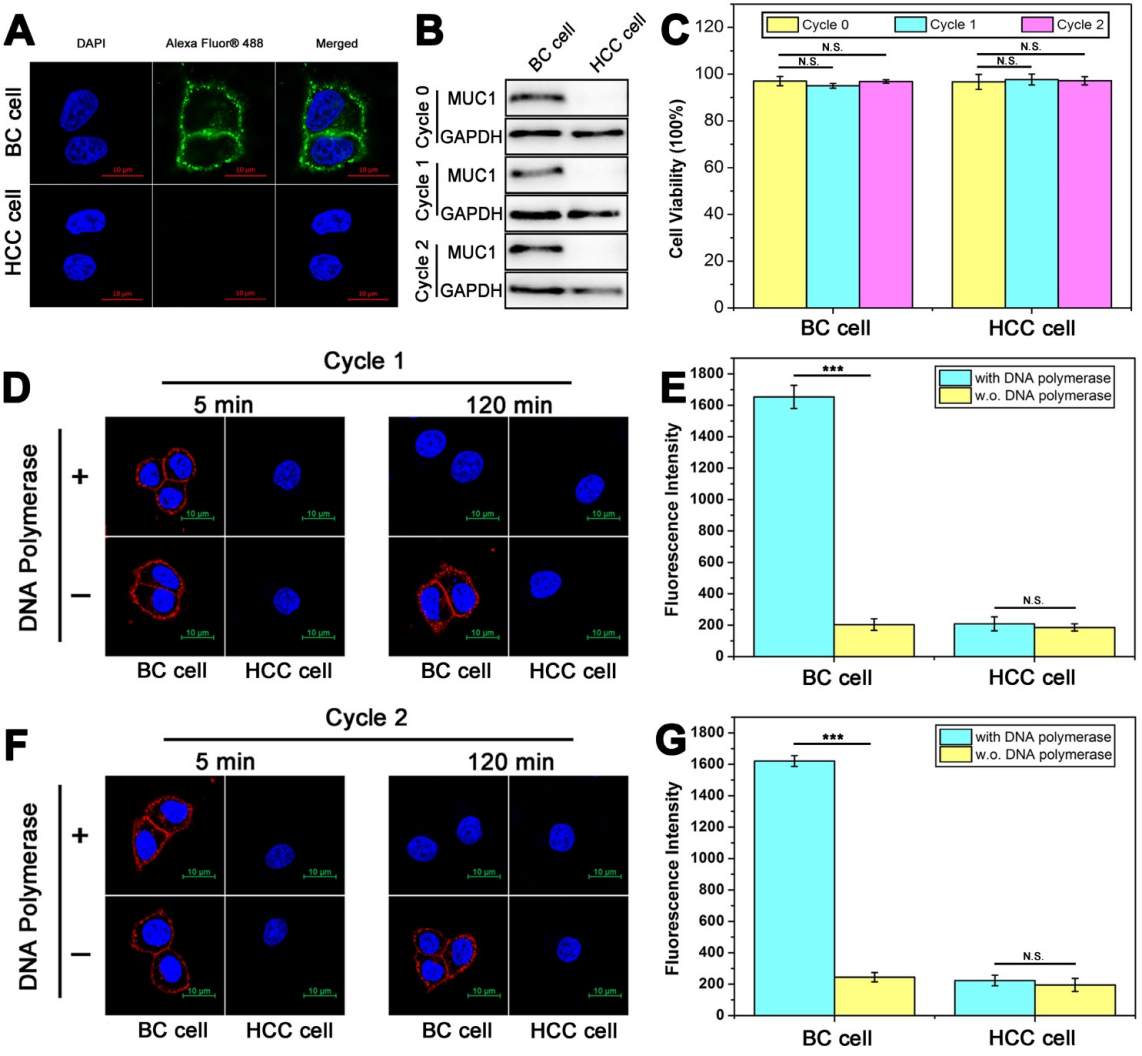
** Nondestructive analysis of breast cancer cells and hepatocellular carcinoma cells that were acquired from volunteer patients and cultured *in vitro*. (A)** Confocal laser scanning microscope images of MUC1 expression with immunofluorescent assay using anti-MUC1 antibody. **(B)** Western blot results of MUC1 expression before (Cycle 0) and after one (Cycle 1) or two (Cycle 2) rounds of DTA analysis. **(C)** CCK-8 analysis of cell viability with 0/1/2 rounds of DTA analysis. **(D-G)** Confocal laser scanning microscope images of MUC1 labeled with Cy5-labeled Apt-Pri in the first round (D) and second round (F) of DTA analysis, and corresponding fluorescent signals of released FAM-labeled molecular beacon after the first round (E) and second round (G) of DTA analysis. Ex: 488 nm, Em: 520 nm. The data represent the mean ± SD (error bars) of triplicate experiments. N.S., no significance. *, p<0.05. **, p<0.01. ***, p<0.001.

To further verify the practical application of DTA-based nondestructive analysis, breast cancer (BC) cells and hepatocellular carcinoma (HCC) cells acquired from *in vivo* tissues were analyzed. MUC1 expression of BC cells and HCC cells was verified first. As shown in Figure 6A and Figure 6B, the results of immunofluorescence and WB showed that BC cells were MUC1-positive while HCC cells were MUC1-negative. Then, two rounds of DTA-based nondestructive analysis were performed. As shown in Figure 6B-6G, there were no significant changes in the expression of MUC1 and cell viability of both BC cells and HCC cells. Moreover, the fluorescence of MUC1 on the cell surface in the confocal images and the fluorescence intensity of the molecular beacon after DTA analysis kept steady in the two rounds of analysis, indicating that the expression of MUC1 on cell membrane is stable during the whole analysis. The above results together revealed that the whole DTA analytical processes do little effect on the expression level of target membrane protein and the growth of the cells, and it is confirmed that this method does not affect cell viability or induce apoptosis. Therefore, the cells can be adopted for further experiments, such as subsequent analysis, culture or drug susceptibility testing of the cells, *etc*. And it may provide a strategy to integrate our DTA analysis further with the membrane analysis or other applications for cell analysis.

## Conclusions

In summary, we have successfully constructed a DTA analysis method based on DNA ternary complex for nondestructive analysis of membrane protein in living cells. In this strategy, DNA ternary complex labeled with fluorophore provides location information of target membrane protein. Subsequently, the extension of Ass-Pri releases DNA ternary complex from target membrane protein and the extension of Apt-Pri produces long chain RCA products in solution for quantification. That is, systematic analysis of location information and expression level of tumor-associated membrane proteins can be achieved based on DTA method. Moreover, repeatable analysis of target membrane protein is achieved. Especially, even after two rounds of imaging and quantitative analysis, the expression level of target membrane protein and the activity of cells are not significantly changed, which indicates the target cells and membrane proteins can be used for further analysis. Therefore, it is believed that this DTA-based method is a superior technique for nondestructive analysis of membrane proteins and is of great value to promote the development of repeatable and in-depth analysis of precious cell samples.

## Supplementary Material

Supplementary figures and table.

## Abbreviations

DTA: dual-terminal amplification
IF: immunofluorescence
WB: western blot
MS: mass spectrometry
DAPI: 4', 6-diamidino-2-phenylindole
DMEM: Dulbecco's modified Eagle's medium
FBS: fetal bovine serum
PFA: paraformaldehyde
CPM: chlorpromazine
BSA: bovine serum albumin
CCK-8: cell counting kit-8
PMSF: phenylmethanesulfonyl fluoride
PVDF: polyvinylidene difluoride
RCA: rolling circle amplification
MB: molecular beacon
BC: breast cancer
HCC: hepatocellular carcinoma
