# Supplementary material for: Nondestructive analysis of tumor-associated membrane protein MUC1 in living cells based on dual-terminal amplification of a DNA ternary complex
Source: Theranostics. 2020 Mar 15;10(10):4410–21. doi: 10.7150/thno.42951 (PMC7150497; doi:10.7150/thno.42951)
Supplement: Supplementary file 1 — Supplementary figures and table. [file thnov10p4410s1.pdf]

Supporting information

Nondestructive analysis of tumor-associated membrane protein MUC1 in living cells based on dual-terminal amplification of a DNA ternary complex

Xiaohao Liu[1,#], Dongsheng Mao[1,#], Guoli Deng[2], Yuchen Song[1], Fan Zhang[1], Shiqi Yang[1], Genxi Li[1], Feng Liu[3,*], Wei Cao[4,*], Xiaoli Zhu[1,*]

[1]Center for Molecular Recognition and Biosensing, School of Life Sciences, Shanghai University, Shanghai 200444, P. R. China

[2]Plant Science Center, School of Life Sciences, Shanghai University, Shanghai 200444, P. R. China

[3]Oncology Department, Shanghai Ninth People's Hospital, Shanghai Jiaotong University, School of Medicine, Shanghai 201900, P. R. China

[4]Department of Oral and Maxillofacial-Head and Neck Oncology, Shanghai Ninth People's Hospital, Shanghai Jiao Tong University, School of Medicine, Shanghai 200011, P. R. China

[#]These authors contributed equally in this work

*Corresponding authors.
E-mail addresses: liufeng@shsmu.edu.cn (F. Liu), caowei561521@shsmu.edu.cn (W. Cao), xiaolizhu@shu.edu.cn (X. Zhu).

Table S1. Details of DNA oligonucleotide sequences.

Name	Sequence (5'→3')	Modification
Apt-Pri	GCAGTTGATCCTTTGGATACCCTGGACCTCACGACCAT TCTGC	5'-Cy5
Ass-Pri	CTAACAATTATCACTGGGTCGTGAGGT	
Loop	GTACGGCAGAATCAGTGATAATTGTTAGAAGAAAAAA AAATCCCAACCCGCCCTACCCTA	Cyclization
Molecular Beacon	CGCTCTCCCAACCCGCCCTAGAGCG	5'-FAM 3'-BHQ
mApt-Pri	ATCTGTATTCATGTATTCTTGTATTACCTCACGACCATT CTGC	5'-Cy5
MUC1 mimic	ATCCAAAGGATCAACTGCCGTAC	5'-FAM

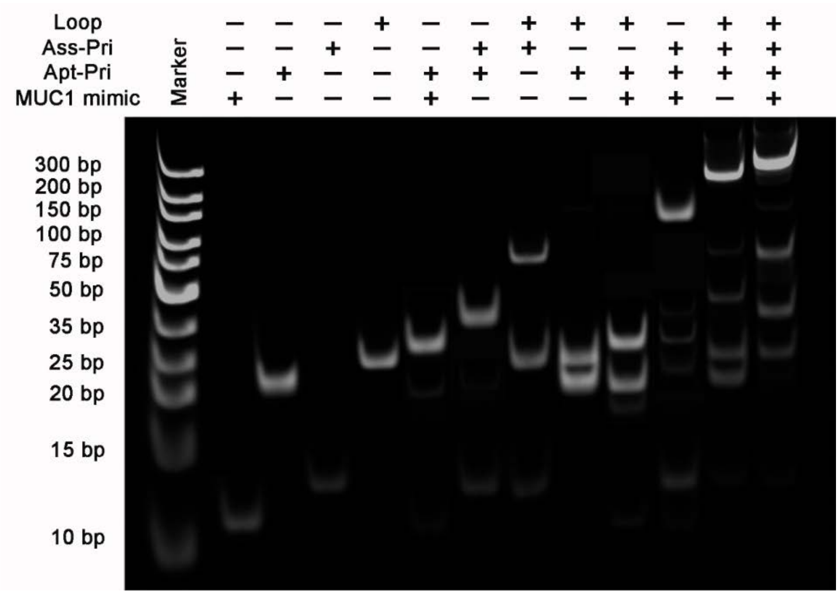

Figure S1. Polyacrylamide gel electrophoretic patterns of the assembly of DNA ternary complex.

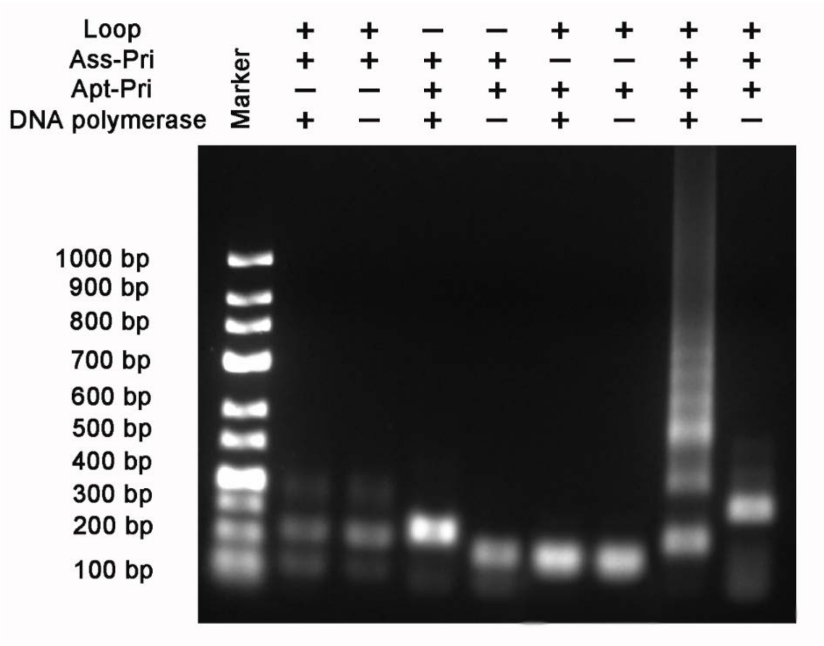

Figure S2. Agarose gel electrophoretic patterns of RCA products with different structures.

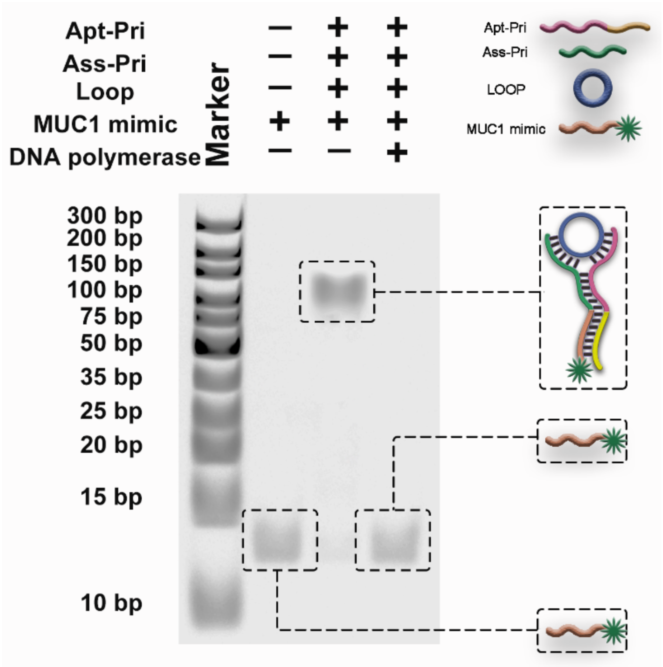

Figure S3. Agarose gel electrophoresis patterns of the release of MUC1 mimic. (Lane 2 to lane 4 are not stained with nucleic acid dye).

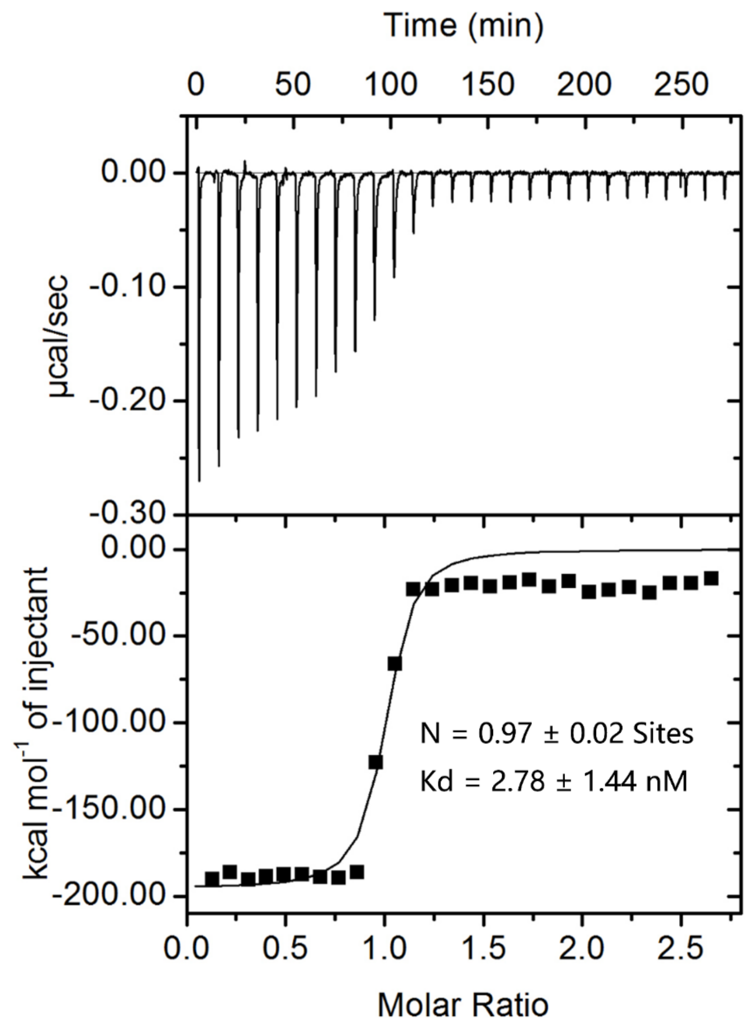

Figure S4. Isothermal titration calorimetry (ITC) analysis of the dissociation equilibrium constant (Kd) of Apt-Pri and MUC1 mimic.

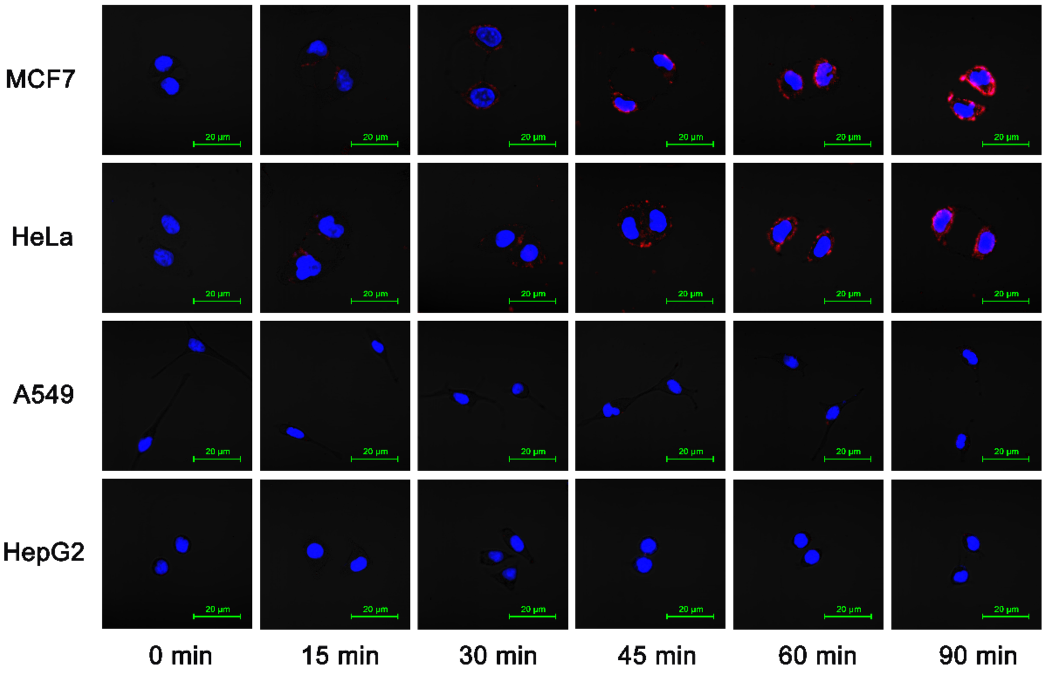

Figure S5. Confocal laser scanning microscopy images of MUC1 labeled with Apt-Pri by various incubated times.

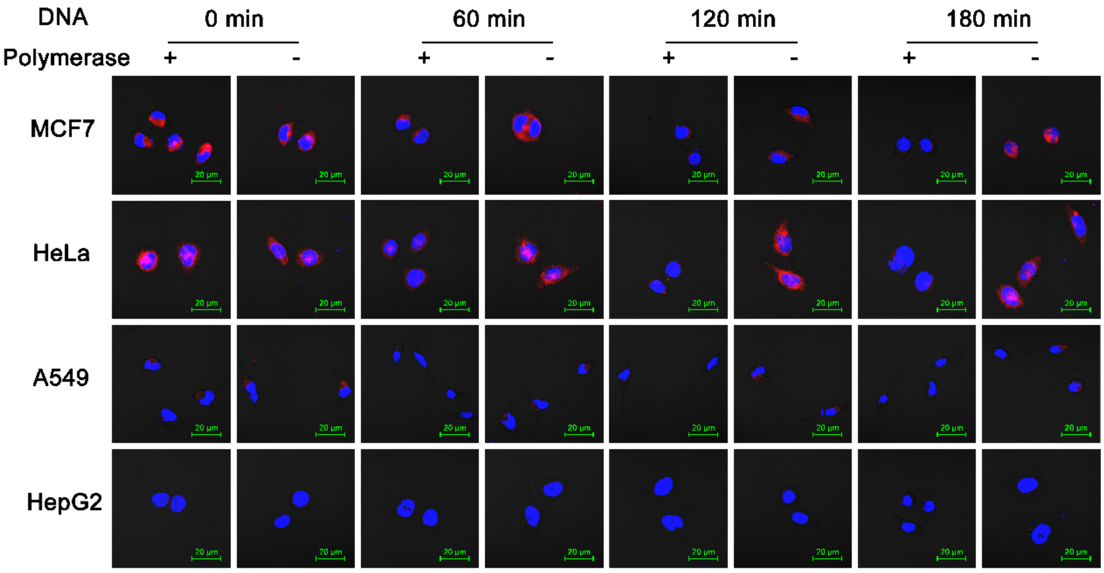

Figure S6. Confocal laser scanning microscope images of Apt-Pri labeled on MUC1 during DTA.

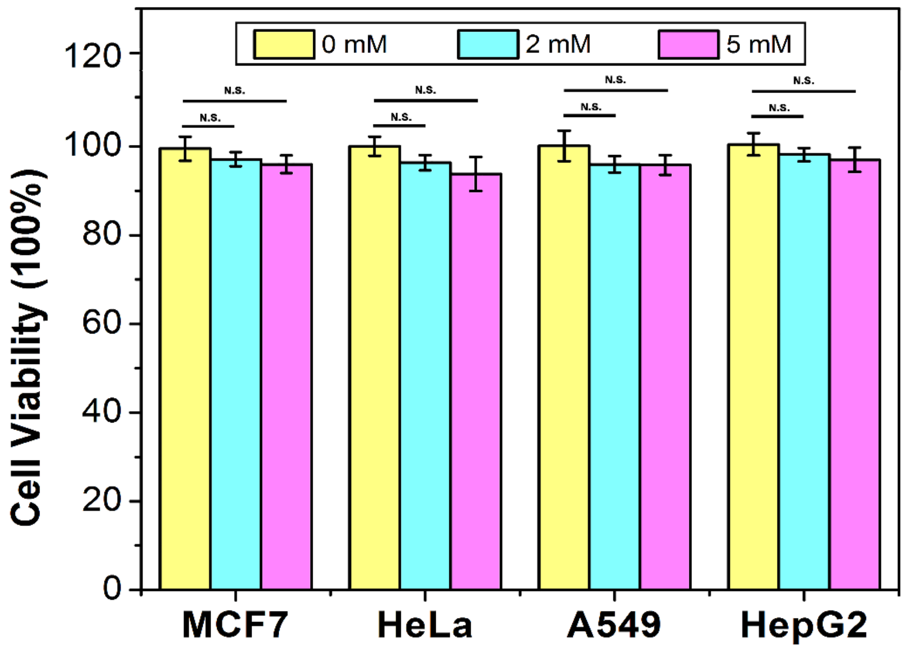

Figure S7. CCK-8 analysis of cell viability. The cells had been pre-treated with various concentrations of benzyl-α-GalNAc for 60 h. The data represent the mean ± SD (error bars) of triplicate experiments. N.S., no significance. *, $p<0.05$. **, $p<0.01$. ***, $p<0.001$.

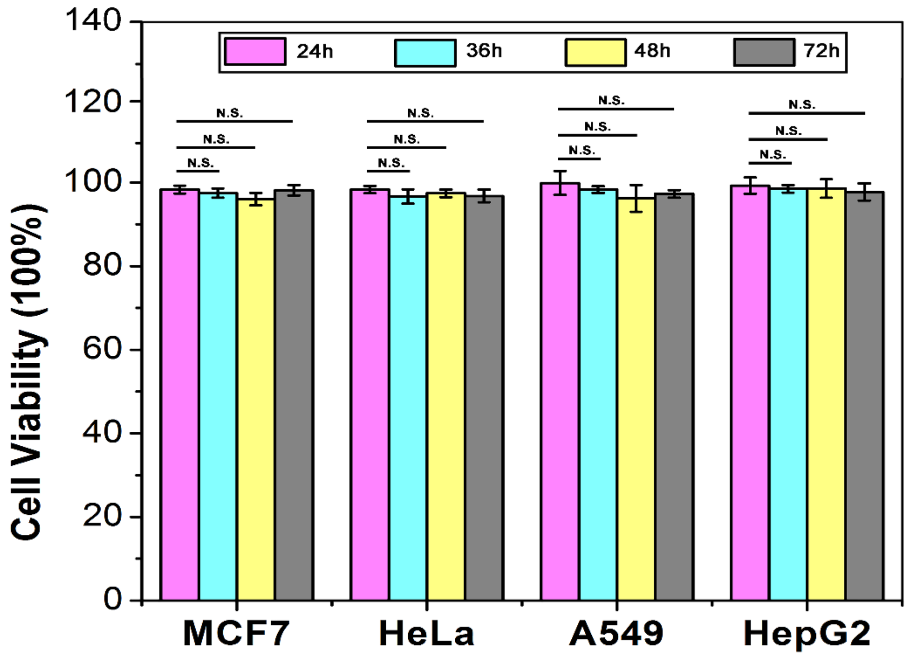

Figure S8. CCK-8 analysis of cell viability. The cells had been pre-analyzed by DTA with two rounds followed by culture for 24-72 hours. The data represent the mean ± SD (error bars) of triplicate experiments. N.S., no significance. *, $p<0.05$. **, $p<0.01$. ***, $p<0.001$.

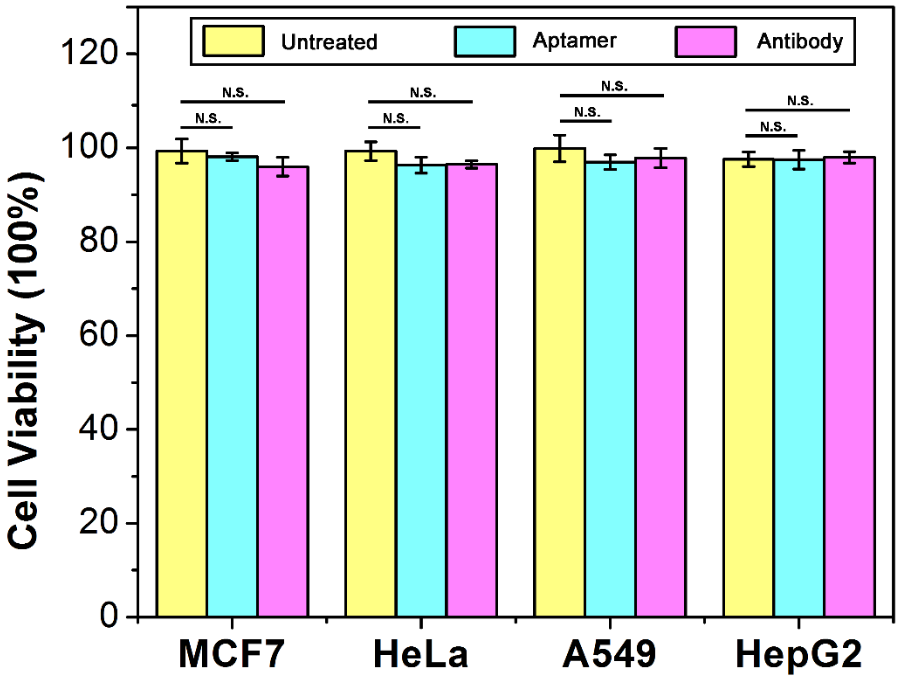

Figure S9. CCK-8 analysis of cell viability. The cells had been pre-incubated with aptamer (Apt-Pri, 150 nM) or Anti-MUC1 antibody (1:500 dilution) for 72 hours. The data represent the mean ± SD (error bars) of triplicate experiments. N.S., no significance. *, p<0.05. **, p<0.01. ***, p<0.001.

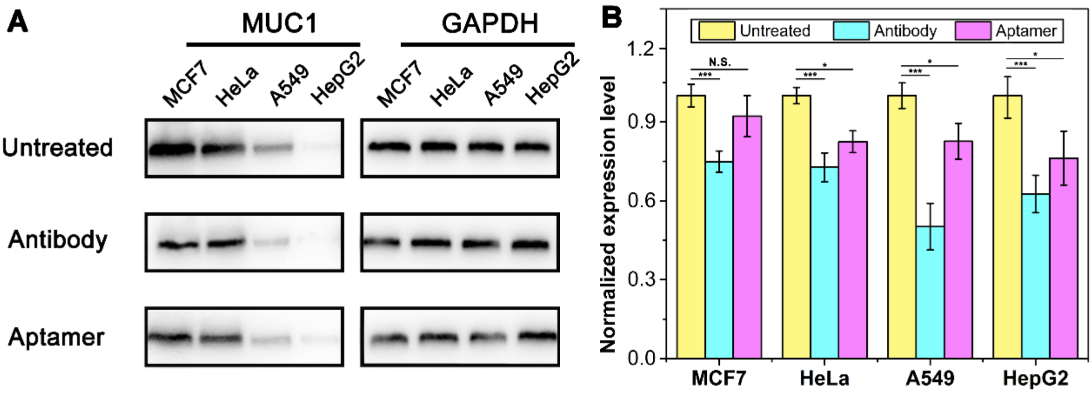

Figure S10. Effect of aptamer and antibody on MUC1 expression in different cell lines. (A) Western blot results and (B) normalized expression level of MUC1 after pre-incubated with aptamer (Apt-Pri, 150 nM) or anti-MUC1 antibody (1:500 dilution) for

72 hours. Normalized data was derived from the gray value of western blot results in Figure S10A. The data represent the mean ± SD (error bars) of triplicate experiments. N.S., no significance. *, $p<0.05$. **, $p<0.01$. ***, $p<0.001$.
